# The Primacy-of-Warmth Effect on Spontaneous Trait Inferences and the Moderating Role of Trait Valence: Evidence From Chinese Undergraduates

**DOI:** 10.3389/fpsyg.2018.02148

**Published:** 2018-11-12

**Authors:** Qing Zhang, Meifang Wang

**Affiliations:** ^1^College of Politics and Public Administration, Shandong Youth University of Political Science, Jinan, China; ^2^College of Elementary Education, Capital Normal University, Beijing, China

**Keywords:** spontaneous trait inferences, spontaneous trait activation, spontaneous trait binding, primacy-of-warmth, valence, warmth, competence

## Abstract

Research has shown that warmth and competence are the fundamental content dimensions underlying social judgment, and warmth judgments are primary. However, the overwhelming majority of research concerning “primacy-of-warmth” rests on trait judgment or lexical recognition, and little attention has been paid to spontaneous trait inferences (STIs) that are made on exposure to trait-implying behaviors. Two studies were performed to examine the primacy-of-warmth effect on STIs and to further explore whether trait valence moderates the effect. Consistent with our expectations, the results of Experiments 1 and 2 (for spontaneous trait activation and spontaneous trait binding, respectively) consistently demonstrated the primacy-of-warmth on STIs. Participants were more likely to draw STIs from behaviors implying warmth traits than those implying competence traits. Moreover, the primacy-of-warmth effect on STIs was moderated by trait valence. In concrete terms, participants were more likely to draw STIs from negative warmth behavioral sentences than negative competence behavioral sentences, whereas participants draw STIs from positive warmth behavioral sentences and from positive competence behavioral sentences equally. An original contribution made by our study is that we obtained the primacy-of-warmth effect on STIs, providing further evidence for the primacy-of-warmth effect in the domain of implicit social cognition.

## Introduction

Previous theory and research have shown that two fundamental content dimensions underlie social judgment (Judd et al., 2005; Abele and Wojciszke, 2007; Fiske et al., 2007). Different names have been given to the two fundamental content dimensions. Researchers refer to communion and agency, warmth and competence, morality and competence, socially and intellectually good or bad, and trust and autonomy, to name just a few (Rosenberg et al., 1968; Fiske et al., 2002; Wojciszke, 2005; Abele and Wojciszke, 2007; Cuddy et al., 2008). The different names of these two dimensions are used in different research areas of psychology. For example, the two dimensions of socially and intellectually good or bad are developed from the multidimensional scaling of traits. Warmth and competence are ubiquitous in research on stereotype and intergroup judgment (Fiske et al., 2002; Judd et al., 2005; Cuddy et al., 2008; Kenworthy and Tausch, 2008). The labels of communion and agency are well established in self and in gender research (Abele, 2003; Abele and Wojciszke, 2007). It is important to note that although so many different labels have been given to these fundamental dimensions, each of these dimensions shares a common core and is operationalized very similarly (Abele and Wojciszke, 2007). The first dimension relates to perceived intent and includes traits such as helpful, warm, aggressive, and selfish, while the second dimension relates to perceived ability and includes traits such as smart, creative, clumsy, and stupid. Like Fiske et al. (1999, 2002) and Chinese language habits, we use warmth and competence to label those content dimensions of social judgment.

Although warmth and competence are two fundamental dimensions underlying social judgment, considerable evidence suggests that the warmth dimension is more important than the competence dimension, a phenomenon referred to as the “primacy-of-warmth” (Abele and Wojciszke, 2007). For example, Wojciszke et al. (1998) demonstrated that participants emphasized warmth over competence when describing the traits that were most indicative of other people (Experiment 1) and when making interpersonal judgments (Experiment 3). Abele and Wojciszke (2007) also found that warmth traits were more important than competence traits when participants were given a number of traits concerning others to rate their importance. Ybarra et al. (2001) showed that subjects recognized warmth traits more quickly than competence traits. Other related findings showed that when drawing trait inferences from faces after an exposure time of 100 ms, participants made more reliable warmth than competence judgments (Willis and Todorov, 2006). Evolutionary psychologists suggest that primacy-of-warmth makes sense because in order to survive, judging others’ intentions (i.e., warmth traits) is more important than judging their capacity to act on their intentions (i.e., competence traits; see Fiske et al., 2007).

However, to our knowledge, the overwhelming majority of research concerning “primacy-of-warmth” rests on trait judgment or lexical recognition, and little attention has been paid to trait inferences that are made on exposure to trait-implying behaviors. Nevertheless, we always see a person’s body and behavior in everyday life instead of this person’s traits. Researchers (Hamilton and Sherman, 1996) argue that inferences from behaviors to traits are one of the principles of information processing in impression formation. These inferences, spontaneous trait inferences (STIs), are often made spontaneously and unintentionally. Some researchers (Uleman et al., 1996; Pomerantz and Newman, 2000) suggest that frequent practice with specific judgments results in their automatization. Previous research showed that participants preferred to deliberately interpret others’ ambiguous behaviors implying both warmth and competence traits in the dimensions of warmth rather than competence (Wojciszke, 1994). Based on previous research on the primacy-of-warmth effect on intent trait inference and the abovementioned theoretical considerations proposed by Uleman et al. (1996), the present research was designed to explore the primacy-of-warmth effect on STIs. It is reasonable to expect that compared to behaviors implying competence traits, participants may make stronger STIs from behaviors implying warmth traits.

Past research on STIs has shown that perceivers not only draw the traits from the actor’s behavior spontaneously (Uleman et al., 1996, 2008; Wigboldus et al., 2003; Yan et al., 2012; Zhang and Wang, 2013) but also ascribe inferred trait constructs to actors (Todorov and Uleman, 2002, 2003, 2004). For example, when reading that “X gives a hand to an old lady and takes her luggage to the train station”, people not only activate “helpful” but also infer that the actor, X, is “helpful” spontaneously. That is, two stages were included in STIs: the first stage is spontaneously activating trait concepts (i.e., trait activation), and the second stage is binding these trait concepts to the actor in memory (i.e., trait binding, see Na and Kitayama, 2011). Thus, it is necessary to explore whether the primacy-of-warmth effect was not only found on spontaneous trait activation but also on spontaneous trait binding.

An additional variable to be considered in this study is the valence of traits. Findings in social perception research suggest that the valence of traits appears to matter substantially in given traits (see Kenworthy and Tausch, 2008), and more weight is given to negative information rather than positive information—a phenomenon referred to as positivity–negativity asymmetry. Some studies (Skowronski and Carlston, 1987; Singh and Teoh, 2000) have shown that such asymmetry specifically holds for traits from the warmth dimension. For example, the study conducted by Skowronski and Carlston (1987) showed that negative behaviors were generally judged as more diagnostic than positive behaviors when the former were warmth dimensions (honesty–dishonesty), but these judgments were reversed for competence dimensions (intelligence–stupidity). Moreover, Ybarra et al. (2001) demonstrated that negative warmth traits were recognized faster than negative competence traits, whereas speed of recognition for positive warmth traits and positive competence traits did not differ significantly. It is worth noting that most studies of an interaction of valence and content dimension rest on trait judgment or lexical recognition. However, relatively little research has explored whether valence moderates the primacy-of-warmth effects on STIs. As such, the present research aimed to further examine whether this is the case.

In this study, two experiments were conducted to explore the primacy-of-warmth effect on STIs and to further explore whether trait valence moderates the effect. In Experiment 1, a probe recognition paradigm was used to examine the primacy-of-warmth effect on spontaneous trait activation and the moderating role of trait valence. Experiment 2 replicated and extended the effects found in Experiment 1 on a later stage of STI – spontaneous trait binding– using a false recognition paradigm.

## Experiment 1

Experiment 1 was designed to examine the primacy-of-warmth effect on spontaneous trait activation by Chinese undergraduates and the moderating role of trait valence. A probe recognition task is well suited to measure the occurrence of spontaneous trait activation (McKoon and Ratcliff, 1986; Ham and Vonk, 2003). In a standard probe recognition task, participants are asked to read many short behavior sentences and respond whether a probe, which follows each of behavior sentences, is contained in the preceding sentence. The task for participants in the probe-recognition paradigm is to indicate whether the probe word existed in the critical sentence. Uleman et al. (1996) suggest that participants have already inferred or not inferred trait properties when they are exposed to behavioral sentences; this is why these are called spontaneous inferences—they happen online, at the time of exposure. Reaction times (RTs) and/or errors in responding to the probe words are evidence that these inferences have been made earlier at exposure. For the experimental behavior sentences, unlike control sentences, the probe words are the trait descriptors that are not in the behavior vignettes but imply the trait related to these behaviors; it is more difficult to produce this correct answer for the experimental sentences (i.e., STIs interfere with task performance). The longer RTs and/or higher errors for the experimental sentences suggest that participants were more likely to have made trait inferences from those sentences compared to control sentences.

In Experiment 1, participants were presented with behavioral sentences implying warmth traits (warmth behaviors) and behaviors implying competence traits (competence behaviors). In line with the expectations mentioned above, we expected that participants made stronger STIs from warmth behaviors than from competence behaviors. Thus, we expected that the correct “no” response is more difficult to trait probes for warmth behaviors than for competence behaviors. Consequently, spontaneous trait activation interferes with the responses, increasing response times (RTs) and/or errors.

It is very difficult to predict whether STIs will affect RTs or error rates. In most studies, the STIs produce effects on either RTs or error rates but not both (Uleman et al., 1996; Ham and Vonk, 2003; Wigboldus et al., 2003). Some researchers have argued that the effect on RTs or error rates may be due to the speed-accuracy trade-off (e.g., Chen et al., 2014). In the probe recognition task, participants are motivated to answer correctly and quickly, but doing both at the same time is most difficult. When participants try to be very fast, they will make more errors, but when participants try to answer very accurately, they start answering more slowly (especially in case of cognitive interference taking place), and thereby, in such cases, effects often are not found on accuracy but are found only on RTs. As such, some researchers have argued that the effects on either RTs or error rates are considered to be sufficient evidence of STIs (Uleman et al., 1996; Todd et al., 2011). Because it is not a priori hypothesis whether the primacy-of-warmth effect on spontaneous trait activation affects RTs or error rates, both of them were analyzed.

### Methods

#### Participants and Design

Forty undergraduates (*M* = 19.71, *SD* = 0.66, range from 18.25 to 22.08; 18 men and 22 women) at Shandong Youth University of Political Science, China, took part in this experiment. All participants were selected in response to an invitational WeChat message. Two experiments were approved by the Institutional Review Board at Educational College of Capital Normal University. Written informed consent was also obtained from participants. The experiment consisted of a 2 (content dimension: warmth vs. competence) × 2 (trait valence: positive vs. negative) repeated-measures ANOVA with all the factors as within-subjects.

#### Stimuli

##### Trait-implying sentences

Based on previous studies (e.g., Abele and Wojciszke, 2007; Abele et al., 2008), three high and three low traits from warmth and competence dimensions (high warmth: helpful, warm and hospitable; low warmth: impolite, selfish and indifferent; high competence: clever, creative and competent; low competence: stupid, clumsy and incompetent) were selected. Correspondingly, 12 behavior sentences implying the above traits were also written. The letter “X” refers to the actor of each of these sentences (Zhang and Wang, 2013; Zhang and Fang, 2016). In a pilot study, participants (*N* = 32) were asked to read the behavioral sentences and indicate on a 7-point scale (1 = not at all, 7 = extremely) to what extent each of these sentences implied the corresponding trait. The order of the sentences was randomized. The results showed that the score for each of the behavior-trait combinations was greater than 5.90, indicating that the trait was implied by corresponding trait-implying behavior. To ensure that each type of sentence equally implied traits, participants’ ratings were submitted to a 2 (content dimension: warmth vs. competence) × 2 (trait valence: positive vs. negative) within-subject ANOVA. The results revealed that no effects were found, *F*s < 1.00, *P*s > 0.05, indicating that the behaviors in the sentences implied traits equally in all conditions. See Table 1 for all of the experimental sentences and traits.

**Table 1:** Ten trait-implying behavior sentences and trait words.

Trait-implying sentence	Trait words
X gives a hand to an old lady and takes her luggage to the train station	Helpful
X enjoys having long conversations with friends.	Warm
X gives the best chocolate to the guest.	Hospitable
X bruises an elder and leaves without apologizing.	Impolite
X hides tasty sugars before the friends arrive.	Selfish
X does nothing when seeing a man with serious injury.	Indifferent
X spends half a minute working out an extremely difficult question.	Clever
X invents a pot that can cook automatically.	Creative
X does a good job no matter how hard the work is.	Competent
X fails to work out such a simple problem the whole morning.	Stupid
X still steps on partner’s feet even after much practice.	Clumsy
No matter what task is assigned by the leader, X cannot complete it.	Incompetent

##### Filler sentences

We selected 36 filler sentences in addition to the 12 experimental sentences to balance the “Yes” and “No” responses. Each of the 24 filler sentences was followed by a probe word in the filler sentence, and each of the other 12 filler sentences was followed by a probe word not in the sentence. Thus, a “Yes” response could be elicited by half of the 48 sentences, while a “No” response could be elicited by the other half.

#### Procedure

The experiment was administered using E-prime software. All instructions and stimuli were displayed on the computer. Each individual participant was seated at a computer and told to complete the memory task. The participant was instructed that a series of sentences would be shown on the computer screen first, and then a word would be displayed after that. The task of the participant was to indicate whether the probe word had appeared in the preceding sentence with speed and accuracy. If the word had appeared in the sentence, the participant should respond with a “Yes” answer. If not, the participant should respond with a “No” answer. Two practice trials were presented to the participant before the actual task as a familiarization task.

The actual task commenced with a stimulus sentence in black letters, which was displayed on the screen for 1500 ms. Then, a probe word in red letters was shown in the center of the screen following a blank screen of 1000 ms. The next trial began after a blank screen was shown for 800 ms once the response was completed. The sequence of the experiment trials was random. The E-prime software recorded the responses and RTs.

### Results and Discussion

#### Reaction Time

In this experiment, erroneous response to the key items did not include the data set. RTs faster than 200 ms and slower than 2000 ms were not included in the data set. In total, 5.01% of the data were disregarded.

Preliminary analyses found no effects due to subject gender, so gender was not considered as a factor in the analyses. The RTs were submitted to a 2 (content dimension: warmth vs. competence) × 2 (trait valence: positive vs. negative) within-subject ANOVA. As indicated in Table 2, a main effect was found for content dimension, *F*(1,39) = 4.25, *p* < 0.05, ${\text{η}}_{\text{p}}^{2}$ = 0.01. This effect indicated that it took participants more time to infer warmth traits from behaviors than competence traits. A main effect was also reliable for trait valence, *F*(1,39) = 11.36, *p* < 0.01, ${\text{η}}_{\text{p}}^{2}$ = 0.23, which indicated that it took participants more time to infer negative traits from behaviors than positive traits. Finally, the interaction of content dimension with trait valence was significant, *F*(1,39) = 5.27, *p* < 0.05, ${\text{η}}_{\text{p}}^{2}$= 0.12. As expected, planned analysis revealed that RTs for different content dimensions of traits did not differ under positive valence conditions, *F*(1,39) = 0.08, *p* > 0.05. However, RTs for warmth probes were longer than for competence probes under negative value conditions, *F*(1,39) = 7.03, *p* < 0.05.

**Table 2:** Mean (SD) reaction times to the key trait probe by content dimension and valence.

Content dimension	Trait valence	
	Positive	Negative
Warmth	852 (162)	963 (214)
Competence	858 (180)	877 (172)

The results of RTs indicated that spontaneous trait activations were stronger from behaviors implying warmth traits (warmth behaviors) compared to behaviors implying competence traits (competence behaviors). Moreover, this primacy-of-warmth effect was moderated by trait valence. In concrete terms, participants made stronger spontaneous trait activations on the basis of warmth behaviors than on the basis of competence behaviors under negative valence conditions. However, none of these effects were found under positive valence conditions.

#### Error Rates

Because errors in this experiment are relatively rare events, it would be reasonable to apply a square root transformation to the data to give less weight to outliers (Wigboldus et al., 2003; see also Ham and Vonk, 2003). Therefore, we first transformed the error data using square roots to reduce the skew. Then, the resulting data were analyzed in a 2 (content dimension: warmth vs. competence) × 2 (trait valence: positive vs. negative) within-subject ANOVA. As indicated in Table 3, this analysis produced a main effect for content dimension, with more errors for warmth than competence sentences, *F*(1,39) = 7.20, *p* < 0.05, ${\text{η}}_{\text{p}}^{2}$ = 0.16. There was also a main effect for trait valence, *F*(1,39) = 8.44, *p* < 0.05, ${\text{η}}_{\text{p}}^{2}$= 0.18. More errors were made to infer negative traits from behaviors than positive traits. The content dimension by valence interaction was not significant, *F*(1,39) = 1.97, *p* > 0.05, ${\text{η}}_{\text{p}}^{2}$ = 0.05.

**Table 3:** Mean (SD) error percentages to the key trait probes as a function of content dimension and valence.

Content dimension	Trait valence	
	Positive	Negative
Warmth	1.67 (0.07)	8.33 (0.15)
Competence	0.00 (0.00)	2.50 (0.08)

The results of error rates demonstrated that perceivers form more spontaneous trait activations from warmth behaviors than from competence behaviors. The trait valence had no influence on this primacy-of-warmth effect. As mentioned above, it is very difficult to predict whether STIs will affect RTs or error rates (Uleman et al., 1996). Significant differences in either RTs or errors are considered to be sufficient evidence of spontaneous inferences (see also Todd et al., 2011; Chen et al., 2014).

In sum, Experiment 1 provided initial support for the primacy-of-warmth effect on STIs. Participants made more spontaneous trait activations from warmth behaviors than competence behaviors. Moreover, the primacy-of-warmth on spontaneous trait activations was moderated by trait valence. In concrete terms, participants made stronger spontaneous trait activations from warmth behaviors than competence behaviors under negative valence conditions. However, none of the effects were found under positive valence conditions.

## Experiment 2

In Experiment 2, our aim was to replicate and extend the effects found in Experiment 1 on a later stage of STIs – spontaneous trait binding– using a false recognition paradigm (e.g., Todorov and Uleman, 2002). In this paradigm, participants read a series of photos of faces paired with trait-implying behavioral sentences (one sentence per face). Later, in a recognition test, participants were presented with a series of face-trait pairs and asked to decide whether they saw the trait in the sentence presented with the face. If trait bindings were spontaneously drawn, false recognition of traits should be higher when the implied traits are paired with the actors’ faces than when implied traits are randomly paired with different faces.

### Methods

#### Participants and Design

Eighty undergraduates (*M* = 18.79, *SD* = 0.70, range from 17.97 to 22.75; 38 men and 42 women) at Shandong Youth University of Political Science, China participated in this experiment. All participants were selected in response to an invitational WeChat message. The design was a 2 (content dimension: warmth vs. competence) × 2 (trait valence: positive vs. negative) × 2 (trial type: systematic pairs vs. random pairs) repeated-measures ANOVA with all the factors as within-subjects.

#### Stimuli

##### Photos

Twenty-four head-and-shoulder photos of Chinese individuals (12 males and 12 females) were selected from a previous study (Yan et al., 2012).

##### Experimental trials

The behavioral sentences that were used for the experimental trials were the same as those of Experiment 1.

##### Filler trials

In addition to the 12 experimental trials, 12 filler trials were developed that also consisted of a behavior description and a trait probe word. Each of these sentences explicitly included the trait. Thus, each of the 12 filler sentences was used to elicit “Yes” responses and prevent the participants from providing only “No” answers.

All 24 sentences were randomly paired with photos of faces.

#### Procedure

Participants completed a false recognition paradigm that was closely modeled after previous research (Todorov and Uleman, 2002). Participants were told that they were taking part in a memory experiment, and the experiment consisted of two related parts. In the first part, they viewed pictures of people accompanied by a sentence describing a behavior performed by that person. In the second part, their memory of this information was tested.

In the first part of the experimental session, each participant was presented with 24 face-behavior pairs. The order of the 24 trial pairs was randomized for each participant. Each face-behavior pair was presented for 10 s with an intertrial delay of 2 s.

The second part of the experimental session consisted of 36 trials; in each trial, the participants would see a photo paired with a trait word. Their task was to indicate whether they had seen this word presented with the face during the first part. The participants were asked to indicate a “yes” answer by pressing the button on the keyboard marked “Yes” and to indicate a “no” answer by pressing the button marked “No.” Each trial was presented until the participant responded, and the delay between trials was 2 s. In 12 of the face-trait pairs, participants were presented with the faces from the filler sentences paired with the traits that had actually been presented during the first phase (filler trials). Another 24 trials, the 12 faces, were correctly paired with implied traits that had not been explicit in the study phase (systematic trials). In the remaining 12 trials, the faces consisting of the same faces as were used for the systematic trials were incorrectly paired with traits implied about a different person (random trials). Participants started with two practice trials to familiarize themselves with the task. The order of the face-trait pairs was randomized for each participant. The computer recorded the response to each test trial. The participants were then thanked and debriefed.

### Results and Discussion

#### Evidence for Spontaneous Trait Binding Formation

The proportion of false recognition of implied traits was calculated separately for the systematic pairs and random pairs for each participant and was used as the dependent variable in subsequent analyses. Preliminary analyses revealed that the effects involving gender were not significant, so gender was not included as a factor in the analyses.

The resulting data were analyzed in a 2 (content dimension: warmth vs. competence) × 2 (trait valence: positive vs. negative) × 2 (trial type: systematic pairs vs. random pairs) within-subject ANOVA. This analysis produced a significant three-way interaction between content dimension, trait valence, and trial type, *F*(l,79) = 10.51, *p* < 0.01, ${\text{η}}_{\text{p}}^{2}$ = 0.12. Planned comparisons showed that in the positive condition, the effect of trial type was significant for warmth dimension, *t*(79) = 3.63, *p* < 0.001, *d* = 0.62: error rates on systematic pairs (*M* = 0.53, *SD* = 0.30) were higher than on random pairs (*M* = 0.35, *SD* = 0.28), and the effect of trial type was also significant for competence dimension, *t*(79) = 3.51, *p* = 0.001, *d* = 0.58: error rates on systematic pairs (*M* = 0.50, *SD* = 0.28) were higher than on random pairs (*M* = 0.34, *SD* = 0.27). In the negative condition, there was a spontaneous trait binding effect for the warmth dimension because the false recognition of trait-face pairs was greater on systematic pairs (*M* = 0.65, *SD* = 0.28) than on random pairs (*M* = 0.29, *SD* = 0.27), *t*(79) = 8.69, *p* < 0.001, *d* = 1.31. The same effect occurred for the competence dimension, with systematic pairs (*M* = 0.50, *SD* = 0.25) showing higher false recognition rates than random pairs (*M* = 0.38, *SD* = 0.29) *t*(79) = 2.67, *p* < 0.01, *d* = 0.44. There is evidence that spontaneous trait binding has been drawn for all conditions (see Todorov and Uleman, 2002).

#### Primacy-of-Warmth Effect on Spontaneous Trait Bindings

To shed light on the primacy-of-warmth effect on spontaneous trait bindings, a false recognition difference score was calculated for each participant by subtracting the mean error rate for random pairs from the mean error rate for systematic pairs (see Wang et al., 2018). This false recognition difference score served as the dependent variable in the following analyses. A 2 (content dimension: warmth vs. competence) × 2 (trait valence: positive vs. negative) within-subject ANOVA revealed a significant effect of content dimension, *F*(l,79) = 11.51, *p* = 0.01, ${\text{η}}_{\text{p}}^{2}$ = 0.13. This difference score was higher for the warmth dimension (*M* = 0.27, *SD* = 0.04) than for the competence dimension (*M* = 0.14, *SD* = 0.04). Most importantly, there was a significant two-way interaction between content dimension and trait valence, *F*(l,79) = 10.51, *p* < 0.01, ${\text{η}}_{\text{p}}^{2}$ = 0.12. After further analysis, we found that the score for the warmth dimension (*M* = 0.37, *SD* = 0.38) was higher than that for the competence dimension (*M* = 0.12, *SD* = 0.40) in the negative condition, but there was no difference between the warmth dimension (*M* = 0.18, *SD* = 0.43) and competence dimension (*M* = 0.16, *SD* = 0.41) in the positive condition.

Overall, Experiment 2 provided evidence that perceivers form more spontaneous trait bindings from warmth behaviors than competence behaviors. Moreover, the primacy-of-warmth effect on spontaneous trait bindings was moderated by trait valence. In concrete terms, participants made stronger spontaneous trait bindings from warmth behaviors than competence behaviors under negative valence conditions. However, none of the effects were found under positive valence conditions.

## General Discussion

To our knowledge, this is the first study to explore the primacy-of-warmth effect on both spontaneous trait activation and spontaneous trait binding and to further explore whether trait valence moderates the effect. Consistent with our expectations, the results of Experiments 1 and 2 (for spontaneous trait activation and spontaneous trait binding, respectively) consistently demonstrated the primacy-of-warmth effect on STIs. Participants were more likely to draw STIs from warmth behavioral sentences than competence behavioral sentences. Moreover, the primacy-of-warmth effect on STIs was moderated by trait valence. In concrete terms, participants were more likely to draw STIs from negative warmth behavioral sentences than negative competence behavioral sentences, whereas there was no difference between the strength of tendency to make STIs from positive competence behavioral sentences and from positive competence behavioral sentences.

Spontaneous inferences of actors’ behaviors by traits help us to survive in world of complex social situations (Wigboldus et al., 2003; Shimizu, 2017). An original contribution made by our study is that we obtained the primacy-of-warmth effect on STIs. According to dual-processing theory, social cognition not only includes explicit assessments that are deliberative and conscious (explicit processing) but also includes implicit components that are automatic and rapid, outside of conscious awareness (implicit processing, see Greenwald and Banaji, 1995; Uleman et al., 2008). Theory and empirical data have shown that implicit processing apparently dissociates from explicit processing. So far, most researchers in the area of primacy-of-warmth effect have relied on explicit measures (i.e., trait judgment), and little attention has been paid to implicit processing. Implicit processing, compared to explicit processing, is much less susceptible to social expectations (Fazio and Olson, 2003). Moreover, since implicit processing occurs in the early stages of social judgment, it may reflect more social and evolutionary significance compared to explicit processing. As such, the primacy-of-warmth effect with explicit measures may be partially interpreted by a social desirability. For example, using explicit ratings, Kenworthy and Tausch (2008) found that warmth traits were rated as having greater stability and accuracy than competence traits. They argue that there is a social desirability in which perceivers believe that actors are not to deviate from ascribed warmth traits (especially for positive traits) rather than competence traits, as the violation of the desirability results in the greatest potential harm or loss. Using STIs that are less affected by social desirability, the present study also found the primacy-of-warmth effect, suggesting that the effect may be a robust phenomenon. It is important to note that the results of the present study provide some preliminary evidence for the primacy-of-warmth effect in the domain of implicit social cognition only. Future studies using other implicit measures, such as the implicit associate test (IAT), to explore the primacy-of-warmth effect in social cognition are needed.

There are at least two possible reasons why the present study found the primacy-of-warmth effect. First, as mentioned in the introduction, the social environment is richer and more unambiguous for warmth information than for competence information (Abele and Wojciszke, 2007; see also Abele and Bruckmüller, 2011). Other related studies have already shown that participants require fewer behavioral instances to infer warmth traits overall than competence traits (Tausch et al., 2007; Ybarra et al., 2008). As such, people may have a lower threshold for trait inferences from behaviors implying warmth traits in comparison with behaviors implying competence traits. As noted in the introduction, practices with trait inferences contributed to the spontaneity of these inferences. Thus, the primacy-of-warmth effect on STIs might be interpreted in terms of the experience that people use more warmth traits than competence traits to interpret behaviors in daily life. Another explanation of the effect is the “perceiving is for doing” view on person perception (Peeters and Czapinski, 1990; Dunning, 2004). That is, warmth qualities are “other-profitable,” which have direct implications for the observer, whereas competence qualities are “self-profitable,” which have direct implications for the actor him or herself. The frameworks provided by “perceiving is for doing” view may mean that, to survive, it is more important to extract the trait meanings from warmth behaviors than from competence behaviors. As such, perceivers might be more likely to draw STIs from warmth behavioral sentences than from competence behavioral sentences. Another issue that deserves comment is that this research first found that the primacy-of-warmth effect on STIs was moderated by trait valence. In contrast, previous studies suggested that the interaction of content dimension and stimulus valence did not emerge on early stages of information processing, such as word recognition, lexical categorization, and valence-based categorization (see Abele and Bruckmüller, 2011), but emerged on the level of intentional social judgments (Skowronski and Carlston, 1987). This incongruent finding in this study may be interpreted by the mechanism of STIs. Although STIs occur automatically at the first stage of person perception (see Ham and Vonk, 2003), it has been suggested that STIs involve controlled attributional processes characterized as deeper, more elaborative activity (Carlston and Skowronski, 1994, 2005). As such, it is not surprising that the primacy-of-warmth effect on STIs was moderated by trait valence in this study.

It is important to note that the present research also fosters our understanding of the inevitability of STIs. Considering that STIs occur in the first stage of impression formation, it is important to examine the mechanism of STIs. One of the central issues of STIs is whether STIs are inevitable. When do observers more strongly infer the other’s traits from their behaviors spontaneously? Some researchers have argued that negativity bias affects STIs. Inferences made from actors’ negative behaviors, which are relatively uncommon and non-normative, tend to be stronger than those made from actors’ positive behaviors, which are more common and less diagnostic. The research by Shimizu (2017) framed the empirical work in terms of negativity bias on STIs. Shimizu (2017) found that STIs that occurred from actors’ negative-trait-implying descriptions were stronger than those that occurred from positive-trait-implying behaviors (see also Carlston and Skowronski, 2005). The findings from the present research suggested that negativity bias on STIs may occur when perceivers form STIs from behaviors implying the corresponding warmth traits but not from behaviors implying the competence traits.

The data we obtained from the present study were interpreted as demonstrating the primacy-of-warmth effect on STIs. One could argue, however, that the trait-implying behavior sentences implied not only the intended trait but also other traits and that these traits may partially account for the findings. For example, higher specificity of negative relative to positive behavior sentences may explain the interactions in both studies with trait valence (positive vs. negative). One way to support this argument empirically is that the consensus on trait implications of negative behaviors should be higher than the consensus for positive behaviors. Due to the careful preselection, we have already shown that the behaviors in the sentences implied intended negative traits to the same degree as the behaviors implied intended positive traits in the stimuli’ section of method in Experiment 1. On the other hand, from a theoretical perspective, multiple concepts, which may be unrelated or even inconsistent, are activated spontaneously when perceivers “see” others’ behaviors (Ham and Vonk, 2003). For example, Todd et al. (2011) found that participants draw the traits and situational properties from others’ behaviors simultaneously and spontaneously. More importantly, participants were not primarily activating one inference and discounting the others (see also Lee et al., 2017). As such, even though multiple concepts were activated from a behavior sentence, other concepts did not affect the activation of the intended trait.

In addition, two additional limitations and future directions are worth noting. One limitation is that the present studies focus only on warmth and competence dimensions. Recently, studies found that the dimension of warmth consists of two components: sociability and morality (Leach et al., 2007; see also Brambilla et al., 2011), and morality is more important when forming impressions of a person (see Goodwin et al., 2014). Future studies separating morality from the warmth dimension may further clarify the primacy effect on STIs. Another limitation is that the primacy-of-warmth effect on STIs was researched in China, a typically collectivistic culture. Previous research has found that collectivistic cultures referred mostly to warmth, whereas individualistic cultures referred mostly to competence (Schwartz, 1992; Wojciszke, 1997; Abele and Wojciszke, 2007). It is unclear whether the primacy-of-warmth effect on STIs we obtained in Chinese societies could be generalized to Western cultures. More cross-cultural research is needed to elucidate the possible variations across cultures in the primacy-of-warmth effect on STIs.

## Ethics Statement

This study was carried out in accordance with the recommendations of the Ethics Committee of Educational College of Capital Normal University with written informed consent from all subjects. All subjects gave written informed consent in accordance with the Declaration of Helsinki. The protocol was approved by the Ethics Committee of Educational College of Capital Normal University.

## Author Contributions

MW designed the experiments. QZ carried out the experiments, analyzed experimental results, and wrote the manuscript.

## Conflict of Interest Statement

The authors declare that the research was conducted in the absence of any commercial or financial relationships that could be construed as a potential conflict of interest.
